# Estimate of Burden and Direct Healthcare Cost of Infectious Waterborne Disease in the United States

**DOI:** 10.3201/eid2708.210242

**Published:** 2021-08

**Authors:** Stephanie DeFlorio-Barker, Abhilasha Shrestha, Samuel Dorevitch

**Affiliations:** US Environmental Protection Agency, Research Triangle Park, North Carolina, USA (S. DeFlorio-Barker);; University of Illinois–Chicago School of Public Health, Chicago, Illinois, USA (A. Shrestha, S. Dorevitch)

**Keywords:** direct healthcare costs, disease burden, disease control strategies, disease transmission, economic burden, healthcare costs, indirect healthcare costs, infectious waterborne diseases, recreational waterborne illnesses, waterborne illnesses, waterborne pathogens

**To the Editors:** We read with interest an article by S.A. Collier et al. (*1*) estimating the economic burden of waterborne illnesses in the United States. Although we found the study noteworthy, the burden estimates differ greatly from those in our 2018 study (*2*) of the economic burden from recreational waterborne illness in the United States. The studies estimated very different numbers of cases: Collier et al. estimated ≈7.1 million total waterborne illnesses, but we estimated ≈90 million recreational waterborne illnesses in untreated water. Collier et al. estimated $3.3 billion in total direct costs from all waterborne illness caused by 17 pathogens, but we estimated $2.9 billion from recreational waterborne illness alone. Both studies used similar methods to address underreporting and underdiagnosis of illness. Key differences between studies include that Collier et al. summarized healthcare costs associated with infections caused by 17 pathogens that might be waterborne, then relied heavily on expert judgment (*3*) to estimate the proportion attributable to water exposure. In contrast, our study used data from large cohort studies of water recreation to estimate the burden from mild and moderate illnesses and outbreak data to estimate the burden from severe illnesses from water recreation. Collier et al. estimated the direct costs of illness, whereas our study estimated both direct and indirect costs (e.g., workplace absence). Enteric pathogens responsible for gastrointestinal symptoms after water recreation are generally not identified in clinical testing (*4*); because Collier et al. used economic burden estimates from waterborne illness based only on 17 pathogens, the study substantially underestimates the overall number of cases and associated economic burden. Future research should consider cohort and outbreak data for treated and untreated recreational water and drinking water to estimate the total economic burden from waterborne illness.
